# Pallidal Structural Changes Related to Levodopa-induced Dyskinesia in Parkinson's Disease

**DOI:** 10.3389/fnagi.2022.781883

**Published:** 2022-05-06

**Authors:** Jinyoung Youn, Mansu Kim, Suyeon Park, Ji Sun Kim, Hyunjin Park, Jin Whan Cho

**Affiliations:** ^1^Department of Neurology, Samsung Medical Center, Sungkyunkwan University School of Medicine, Seoul, South Korea; ^2^Neuroscience Center, Samsung Medical Center, Seoul, South Korea; ^3^Department of Artificial Intelligence, Catholic University of Korea, Bucheon, South Korea; ^4^Department of Biostatistics, Soonchunhyang University Seoul Hospital, Soonchunhyang University College of Medicine, Seoul, South Korea; ^5^Center for Neuroscience Imaging Research, Institute for Basic Science, Suwon, South Korea; ^6^School of Electronic and Electrical Engineering, Sungkyunkwan University, Suwon, South Korea

**Keywords:** dyskinesia, levodopa-induced dyskinesia, basal ganglia, globus pallidus, pallidum, Parkinson's disease

## Abstract

**Background:**

Despite the clinical impact of levodopa-induced dyskinesia (LID) in Parkinson's disease (PD), the mechanism, especially the role of basal ganglia (BG), is not fully elucidated yet. We investigated the BG structural changes related to LID in PD using a surface-based shape analysis technique.

**Methods:**

We recruited patients with PD who developed LID within 3 years (LID group, 28 patients) and who did not develop it after 7 years (non-LID group, 35 patients) from levodopa treatment for the extreme case-control study. BG structure volumes were measured using volumetry analysis and the surface-based morphometry feature (i.e., Jacobian) from the subcortical surface vertices. We compared the volume and Jacobian of meshes in the regions between the two groups. We also performed a correlation analysis between local atrophy and the severity of LID. Additionally, we evaluated structural connectivity profiles from globus pallidus interna and externa (GPi and GPe) to other brain structures based on the group comparison.

**Results:**

The demographic and clinical data showed no significant difference except for disease duration, treatment duration, parkinsonism severity, and levodopa equivalent dose. The LID group had more local atrophies of vertices in the right GPi than the non-LID group, despite no difference in volumes. Furthermore, the LID group demonstrated significantly reduced structural connectivity between left GPi and thalamus.

**Conclusion:**

This is the first demonstration of distinct shape alterations of basal ganglia structures, especially GPi, related to LID in PD. Considering both direct and indirect BG pathways share the connection between GPi and thalamus, the BG pathway plays a crucial role in the development of LID.

## Introduction

Considering Parkinson's disease (PD) is a neurodegenerative disorder with progressive dopaminergic degeneration, the administration of levodopa, a dopamine precursor, is the most effective treatment for PD. However, chronic levodopa medication could cause disabling motor complications, especially levodopa-induced dyskinesia (LID), which affects the quality of life of patients with PD. Additionally, almost half of patients could develop dyskinesias after 5 years of treatment, and the majority of patients after 10 years (Manson et al., 2012).

Although the mechanism of LID development is not completely understood, presynaptic dopaminergic denervation and chronic pulsatile stimulation of dopamine receptors have been considered to be associated with its development (Calabresi et al., 2010). However, the time of onset and LID severity are highly heterogeneous among patients (Hely et al., 2005). These variable presentations could be explained by the different involvement of relevant brain structures associated with LID. Studies reported that morphological changes were strong biomarkers with wide applications in various neurodegenerative diseases (Ment et al., 2009; Turcano et al., 2018). LID development could be delayed with medication and LID severity could be effectively controlled with deep brain stimulation (Follett, 2004), thus it is important to find the brain structure changes with LID in patients with PD. However, the morphological substrate of LID in patients with PD remains underexplored.

Surface shape analysis is a sensitive and quantitative technique to detect structural changes in the subcortical nuclei in patients with PD (Lee et al., 2014; Menke et al., 2014). Therefore, the present study aimed to explore the structural changes in the basal ganglia, connected to LID development in patients with PD, using a volumetry, surface-based shape analysis technique, and connectivity analysis.

## Methods

### Subjects and Study Design

This study was approved by the Institutional Review Board of the Samsung Medical Center, Seoul, Korea (IRB #2012-10-102-017), and each patient provided informed consent to participate. All methods were carried out in accordance with the relevant guidelines and regulations.

For this extreme case-control study design (Salim et al., 2014), we enrolled patients with PD who developed LID within 3 years (LID group) and who did not develop it after 7 years from starting levodopa treatment (non-LID group) at the Movement Disorders Clinic, Samsung Medical Center, Seoul, from March 2013 to December 2016. PD was diagnosed according to the UK Brain Bank Criteria for the diagnosis of PD (Hughes et al., 1992). Subjects with any of the following were also excluded: (1) structural brain lesions, including stroke, tumor, trauma, or white matter changes (age-related white matter change score ≥ 2 on brain MRI) (Wahlund et al., 2001); (2) other known neurodegenerative diseases or psychiatric disorders requiring medications; (3) other diseases, including symptomatic neuropathy, or musculoskeletal problems, that affected parkinsonism.

For clinical assessments, the Unified Parkinson's Disease Rating Scale (UPDRS) part 3, H&Y stage, Unified Dyskinesia Rating Scale (UDysRS), and the Korean version of the Montreal Cognitive Assessment (K-MoCA) were evaluated in all the recruited subjects with PD (Lee et al., 2008). UPDRS part 3 score was divided into 4 sub-scores for tremor, bradykinesia, rigidity, and axial symptom (Diederich et al., 2003). Levodopa equivalent dose (LED) was calculated based on previous literature (Tomlinson et al., 2010). Total LED was divided into 3 groups, which were the levodopa and catechol-O-methyltransferase (COMT) inhibitor, dopamine agonist, and others.

### MRI Acquisition

We collected T1-weighted MRI (T1-MRI) and diffusion-weighted MRI (dMRI) data using a 3-T MRI scanner (Philips 3-T Achieva, Best, the Netherland). The T1-MRI was acquired with the following acquisition parameters: sagittal slice thickness, 1 mm; contiguous slices with 50% overlap; no gap; repetition time (TR), 9.9 ms; echo time (TE), 4.6 ms; flip angle, 8°; matrix size of 240 × 240 pixels, which was reconstructed to 480 × 480 over a field of view (FOV) of 240 mm. The dMRI was acquired with the following acquisition parameters: TR/TE = 5,900/88 ms, 2 mm^3^ isotropic resolution; 72 contiguous slices, two-fold acceleration, axial–oblique aligned along the anterior-posterior commissure, and diffusion-weighting along 64 gradient directions with a b-value of 1,000 s/mm^2^.

### Volumetric and Shape Analysis

The brain morphometry was evaluated on the left and the right caudate nucleus, putamen, globus pallidus, and thalamus in terms of volume and Jacobian determinant. The volume of each structure was computed using FreeSurfer (version 6; Athinoula A. Martinos Center at the Massachusetts General Hospital, Harvard Medical School, MA, USA) (Fischl, 2012). We normalized the volume by dividing the calculated volume by the intracranial volume. The Jacobian determinant was used to measure the ratio of surface dilation between a given subject and the template in the region. The Jacobian determinant was computed according to the protocol set by the ENIGMA consortium (Gutman et al., 2012, 2013). Briefly, we obtained the subject-specific segmentation of the subcortical structures and then applied the “Medial Demons” framework to register subcortical shapes onto the pre-specified template (Roshchupkin et al., 2016). The meshes for the eight basal ganglia structures of each subject were defined on the template space. Finally, we computed the natural logarithm of the Jacobian determinant (referred to as *Jacobian* hereafter) that represented the ratio of surface dilation between the given subjects with respect to the template.

### Connectivity Analysis

**Figure 1:**
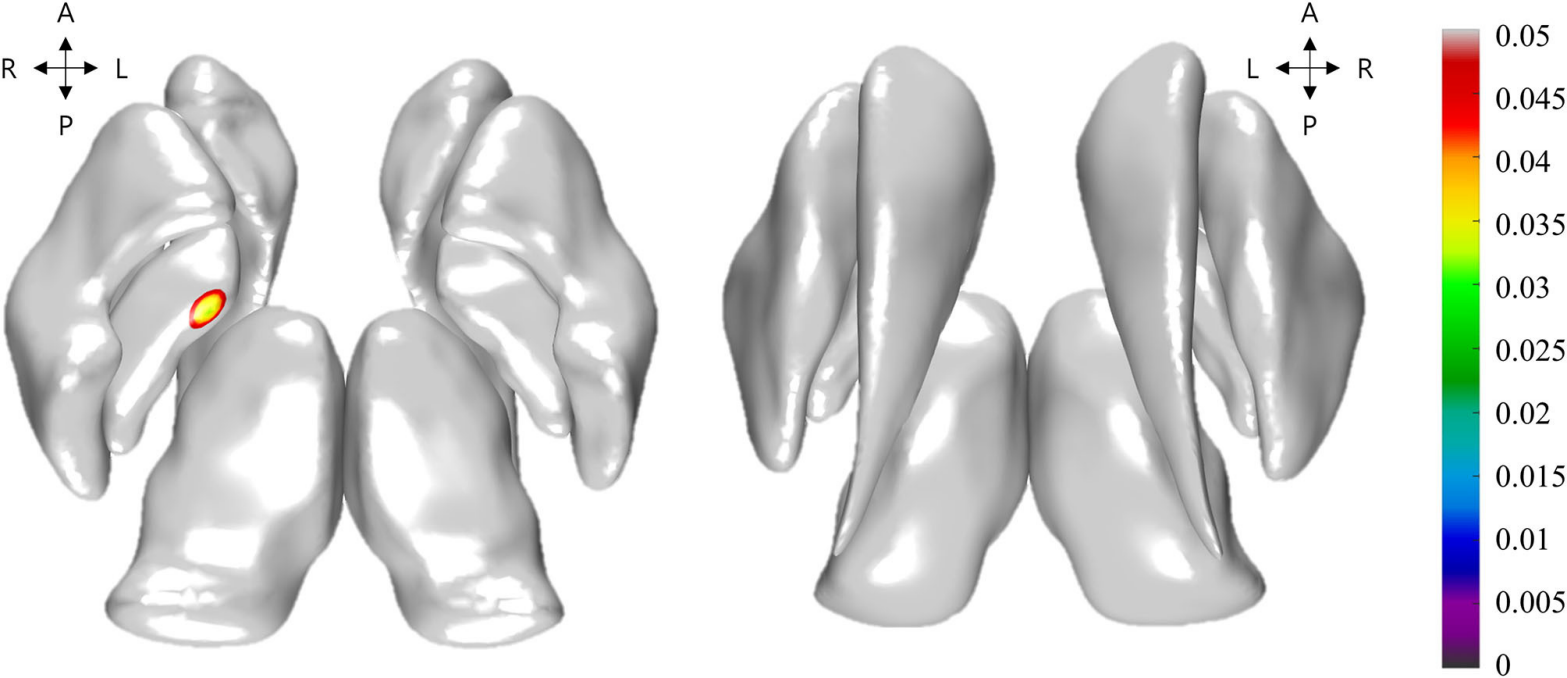
Illustration of the group-wise differences of Jacobian between levodopa-induced dyskinesia (LID) group and non-LID group. Non-gray color regions denote the vertices with significantly different Jacobian. The color bar shows the corrected *p*-values for the statistical test. The left and right subfigures denoted the top axial view and bottom axial view, respectively.

Additionally, based on the initial results of basal ganglia structures between two groups in the present study (Figure 1), we chose globus pallidus and compared the structural connectivity profiles of globus pallidus to other brain structures between LID and non-LID groups. The structural connectivity was computed using the probabilistic tractography algorithm implemented in FSL (probtrackX) (Behrens et al., 2007). Briefly, we performed pre-processing steps using FSL software, which were as follows: intensity normalization, distortion correction, eddy current correction, and head motion correction. Then, we estimated the fiber orientation for each voxel from dMRI with the multi-shell-spherical deconvolution toolbox (bedpostx) (Behrens et al., 2007; Jbabdi et al., 2012). We estimated fiber streamlines for every voxel using probtrackX and mapped them onto the brain regions. The 83 brain regions were defined based on the Desikan-Killany atlas and we manually divided the globus palladium regions into two sub-regions (e.g., globus pallidus interna and externa), yielding a total of 85 brain regions. Finally, we computed and compared the structural connectivity profiles of left and right globus pallidus interna and externa (GPi and GPe) between the LID and non-LID groups.

### Statistical Analysis

The demographic and clinical variables were compared between LID and non-LID groups using an unpaired Student's *t*-test or the Mann-Whitney U test for continuous and ordinal variables, while Pearson's chi-square test or Fisher's exact test were used to determine categorical variables. We rejected the null hypotheses of no difference if *p*-values were < 0.05. These statistical analyses were conducted using commercially available software (PASW for Windows, version 18; SPSS, Chicago, IL, USA). For the volume and structural connectivity profile analysis, we performed two-sample student's *t*-tests for identifying the group difference between LID and non-LID groups, and the Bonferroni correction was conducted for correcting the multiple comparisons issue.

Group-wise differences of the *Jacobian* in the basal ganglia structures between the LID group and non-LID groups were assessed with non-parametric permutation tests adjusted for age, disease duration, treatment duration for levodopa medication, LED for levodopa and COMT inhibitor, and dopamine agonist, and UPDRS part 3 sub-scores (Nichols and Holmes, 2001). We performed the permutation tests by randomly assigning LID and non-LID groups 10,000 times. If the real difference of *Jacobian* did not belong to the 95% of the null distribution derived from the permutations, it was deemed significant and the multiple comparisons were corrected by false discovery rate (FDR) correction (*p* < 0.05) (Boca and Leek, 2018). The correlation analysis was performed to detect potential links between the *Jacobian* of basal ganglia structures and the UDysRS score. These statistical analyses were performed with MATLAB (The MathWorks Inc., Natick, MA, USA) (MATLAB and Statistics Toolbox Release, 2020).

## Results

### Demographic and Clinical Features of LID and Non-LID Groups

We enrolled 63 non-demented right-handed subjects, among which 28 patients were in the LID group and 35 patients were in the non-LID group. Table 1 presented the demographic and clinical data of each group. There were no significant differences in demographic data between the two groups. In terms of clinical data, the non-LID group demonstrated significantly longer disease duration, higher tremor, and lower bradykinesia sub-score than the LID group. For medication, the LID group took more medication (total LED and LED for levodopa and COMT inhibitor) than the non-LID group.

**Table 1:** Demographic information of PD with and without LID.

	**Early-LID group**	**Non-LID group**	***p*-value**
Number of subjects	28	35	–
Onset age (years)	52.3 ± 9.2	53.1 ± 6.2	0.661
Disease duration (years)	6.4 ± 2.4	11.4 ± 3.2	<0.001
Treatment duration for antiparkinsonian medication (month)	64.9 ± 30.1	117.9 ± 38.4	<0.001
Treatment duration for levodopa medication (month)	56.3 ± 28.9	85.2 ± 57.5	0.078
Sex (Male/Female)	12/16	17/18	0.800
Symptom-dominant side, right/left	14/14	19/16	0.803
UPDRS part 3			
Tremor	2.6 ± 2.8	3.8 ± 2.4	0.024
Bradykinesia	14.4 ± 5.1	7.6 ± 8.3	0.002
Rigidity	5.8 ± 2.5	4.9 ± 3.2	0.085
Axial symptoms	7.8 ± 2.9	7.8 ± 3.7	0.838
Total score	30.6 ± 9.0	24.2 ± 12.7	0.009
HY stage	2.3 ± 0.6	2.2 ± 0.6	0.689
K-MoCA	26.1 ± 3.3	25.8 ± 2.6	0.509
UDysRS	19.8 ± 13.7	0	<0.001
Levodopa equiva lent dose			
Levodopa + COMT inhibitor (mg/day)	631.1 ± 246.6	419.5 ± 252.3	0.001
Dopamine agonist (mg/day)	166.5 ± 86.4	207.6 ± 149.7	0.469
Others (mg/day)	112.5 ± 131.7	107.4 ± 102.7	0.699
Total dose (mg/day)	910.2 ± 337.5	734.5 ± 305.3	0.009

*UPDRS, Unified Parkinson's Disease Rating Scale; H&Y, Hoehn and Yahr score; K-MoCA, Korean-Montreal Cognitive Assessment; UDysRS, Unified Dyskinesia Rating Scale; LED, levodopa equivalent doses*.

### Comparison of Basal Ganglia Structures Between LID and Non-LID Groups

The volumes of basal ganglia structures were illustrated in Table 2. There was no difference in the volume of basal ganglia structures between the two groups. However, the LID group had significant local atrophy in the right GPi (32 vertices with mean corrected *p* =0.041 ± 0.006 in mean ± *SD*) than the non-LID group (i.e., −0.223 ± 0.164 of Jacobian for LID group; −0.178 ± 0.131 of Jacobian for the non-LID group), as shown in Figure 1.

**Table 2:** Group-wise differences in the volumetric features of eight subcortical regions.

	**Volume (mm** ^**3**^ **)**		***p*-value**
	**LID group**	**Non-LID group**	
Left thalamus	7136.2 ± 797.8	7068.0 ± 932.5	0.761
Right thalamus	6742.9 ± 671.5	6706.4 ± 838.1	0.852
Left caudate	3272.0 ± 564.8	3227.9 ± 431.8	0.728
Right caudate	3331.1 ± 459.2	3367.0 ± 470.8	0.761
Left putamen	4518.7 ± 518.9	4432.2 ± 499.1	0.503
Right putamen	4481.2 ± 455.8	4420.7 ± 504.3	0.619
Left pallidum	1919.0 ± 223.3	1961.5 ± 250.7	0.478
Right pallidum	1875.7 ± 232.3	1906.9 ± 261.2	0.617

### Correlation of the Basal Ganglia Local Atrophy With UDysRS Score

For correlation analysis, we controlled age, disease duration, treatment duration for levodopa medication, LED (levodopa and COMT inhibitor, and dopamine agonist), and UPDRS part 3 sub-scores. There were no basal ganglia areas showing local atrophy significantly correlated with the UDysRS score (Supplementary Figure).

### Comparison of Connectivity From Globus Pallidus to Other Brain Regions

Based on the results of local atrophy in basal ganglia structures, we compared the structural connectivity profiles of globus pallidus to other brain structures. The LID group demonstrated significantly reduced connectivity between left GPi and left thalamus compared to the non-LID group (0.000002 for the LID group and 0.00068 for the non-LID group, corrected *p* =0.017) (Supplementary Table).

## Discussion

This is the first study to the best of our knowledge to explore the distinct shape alterations of basal ganglia structures in patients with PD having with and without LID. We found the local atrophy in right GPi and reduced connectivity between left GPi and thalamus in the LID group compared to the non-LID group. Based on our results, we suggest GPi as a key brain structure related to LID. The present study revealed discrepant results from shape and connectivity analyses, local atrophy in the right pallidum, and reduced connectivity in the left pallidum. In our study, there was no difference in the symptom-dominant side between the two groups. Previous studies using imaging analysis also demonstrated asymmetric results (Herz et al., 2014; Cerasa et al., 2015; Farre et al., 2015), and there is still no consensus about the laterality in LID development.

Previous studies focusing on LID-related anatomical abnormalities showed structural alterations mainly in the cortical structures (Cerasa et al., 2011, 2013). Cortical thickness analysis and voxel-based morphometry revealed increased cortical thickness and gray matter volume in the frontal cortex in patients with LID compared to those without LID. Basal ganglia are structures directly involved in PD, and also with wide efferent and afferent connections with the frontal lobe. Therefore, there would be changes in basal ganglia structures between the aforementioned cortical changes and nigral degeneration in PD. However, unlike the anatomical changes in the cortical structures, those in the basal ganglia were not fully investigated. A previous study with the PD rat model revealed the hypertrophy of medial GPi and substantia nigra reticulata with levodopa treatment unlike our results (Tomiyama et al., 2004). However, this study focused on the structural changes related to levodopa treatment, not LID, and did not compare between PD models with and without LID.

Additionally, various hypotheses, including pre-synaptic and post-synaptic changes in the basal ganglia, are suggested for LID in PD (Iravani and Jenner, 2011; Phillips et al., 2016), but the main mechanism underlying LID is pulsatile stimulation of the striatal postsynaptic receptors. In patients with PD, dopaminergic modulation of the striatal activity and compensatory mechanism are already impaired; therefore, exogenous administration of repeated doses of levodopa induces molecular and neurophysiological changes (Calabresi et al., 1993), and abnormal firing pattern of the basal ganglia neuron (DeLong, 1990). However, it is difficult to evaluate the changes in subcortical nuclei, including basal ganglia structures. Previous studies used volumetric techniques and failed to find a decrease in the volume or density in the subcortical nuclei even between patients with PD and normal control (Messina et al., 2011). Similarly, we also performed the volumetric analysis of basal ganglia structures and could not find any significant differences in basal ganglia volumes. Surface-based morphometry can provide novel information that cannot be obtained with conventional volumetry and voxel-based analysis (Veldsman, 2017). We adopted the determinant of Jacobian as it provides a compact summary of the surface shape at the regional level compared to using the vertex meshes directly (Veldsman, 2017). Thus, surface-based morphometry could be well-suited to quantify the complex local shape of basal ganglia and we could demonstrate local atrophy of the GPi in patients with PD who developed LID using surface-based morphometry.

Local shape atrophies in the GPi of the LID group might reflect the complex and integrated impairment in these networks. It is underpinned by reduced connectivity between the GPi and thalamus in the LID group compared to the non-LID group. The connection between GPi and the thalamus is a common pathway involved in both direct and indirect pathways, thus the imbalance in GPi between two pathways could be related to LID (Barroso-Chinea and Bezard, 2010). Additionally, these output nuclei of basal ganglia also project connections mainly to the frontal lobe, where the structural difference was reported in a previous study among patients with PD who developed LID (Cerasa et al., 2011, 2013). Therefore, based on our results and previous studies, the output nuclei of basal ganglia (GPi and thalamus) play a crucial role in the development of LID in patients with PD, and these areas could be effective targets for the management of LID. Deep brain stimulation in the GPi has direct suppression effects on LID in patients with PD (Follett, 2004), and fibroblast transplantation at GPi also improved LID in the primate model (Singh et al., 2015).

However, it is difficult to explain the laterality of GPi in shape and connectivity analysis. We used multi-model imaging analysis, and the difference in methodology could have also affected our results. The local atrophy of right GPi was from shape analysis with T1-MRI while reduced connectivity between left GPi and thalamus from tractography with dMRI. However, both imaging analyses demonstrated that changes in GPi were related to LID in patients with PD, thus we suggested the importance of GPi in LID development in patients with PD.

In our study, there were significant differences, including age, disease duration, UPDRS part 3 score, and LED between LID and non-LID groups. Various clinical variables, including early age at onset, non-tremor dominant subtype, and levodopa dose, were already reported as risk factors for LID in patients with PD (Tran et al., 2018; Lee et al., 2019). In accordance with previous studies, LID groups showed earlier age at onset, less tremor sub-score of UPDRS part 3, and higher levodopa dose in our study. As well as the clinical factors associated with LID, the disease itself and even medications that could cause structural changes in patients with PD. To overcome the possible confounding factors, we controlled these variables, including age, disease duration, treatment duration, medications, and motor subtypes, in the imaging analysis. Therefore, if we control fewer clinical variables in imaging analysis, we might find more brain areas related to LID. Additionally, LID is usually present in patients with advanced PD and these patients could show a wide clinical spectrum. In the present study, we tried to eliminate possible confounding effects from other symptoms, because various brain structures could reflect all of these symptoms, as well as LID.

Our study has several strengths. To maximize the shape differences between the patient with and without LID, we used the extreme case-control study design: the earliest development of LID for the LID group and the longest-surviving control from LID for the non-LID group. This is a research design that is one of the most used methods to efficiently estimate a model with less sample size and costs. Although the duration of the disease was shorter in the LID group, the pallidal atrophy was more severe in the LID group. This method improves the efficiency as compared to the standard study design. Besides, we used shape analysis, which offers an intuitive and powerful means of quantifying anatomy in the context of brain imaging. However, this study also has some limitations. The cross-sectional study design made the assessment of time-related changes difficult. A longitudinal design will be necessary to confirm these shape alterations as the dynamic components of LID development. Although there are several advantages of computing fiber tractography based on probabilistic tractography, it may lead to the false-positive fiber bundle estimation due to its high sensitivity in low FA voxels. Hence, this analysis should be further confirmed with different tractography algorithms with independent replications to reduce false discoveries. In addition, we enrolled a small-sized sample; however, even with the sample size, we were able to identify the basal ganglia structures associated with LID, using the extreme case-control study design. Lastly, early-LID and non-LID groups in our study showed different clinical characteristics, thus it is impossible to compare the two groups directly. PD is a heterogeneous disorder with a wide clinical spectrum and the progression rate could vary depending on the subtypes of PD. We adjusted as many clinical variables as possible, including disease duration, treatment duration medication, and UPDRS part 3 sub-scores, to minimize the possible confounding effect from subtypes associated with different progressions.

In conclusion, our study demonstrated that local atrophy of GPi and reduced connectivity with the thalamus were related to LID in patients with PD. This is the first study to demonstrate distinct shape alterations of basal ganglia structures related to LID and our results emphasized the role of basal ganglia pathways in the development of LID.

## Data Availability Statement

The data analyzed in this study is subject to the following licenses/restrictions: the data that support the findings of this study are available on request from the corresponding authors (JC or HP). The data are not publicly available due to research participant privacy/consent. Requests to access these datasets should be directed to JC, jinwhan.cho@samsung.com.

## Ethics Statement

The studies involving human participants were reviewed and approved by the Institutional Review Board of the Samsung Medical Center, Seoul, Korea (IRB #2012-10-102-017). The patients/participants provided their written informed consent to participate in this study.

## Author Contributions

JY, JK, MK, HP, and JC contributed to the concept and design. JY, JK, and JC contributed to data acquisition. MK, SP, and HP contributed to the data analysis. JY, SP, JK, and MK contributed to the interpretation of data. JY, MK, SP, HP, and JC contributed to substantive revision. All authors contributed to the article and approved the submitted version.

## Funding

This study was supported by the Institute for Basic Science (Grant Number IBS-R015-D1), National Research Foundation of Korea (NRF-2020M3E5D2A01084892 and NRF-2020R1A6A3A03038525), Ministry of Science and ICT (IITP-2020-2018-0-01798), IITP grant funded by the AI Graduate School Support Program (2019-0-00421), ICT Creative Consilience program (IITP-2020-0-01821), and the Artificial Intelligence Innovation Hub program (2021-0-02068).

## Conflict of Interest

JY received speaker's honoraria from SK chemicals, and research support from Medtronic and Boston Scientific. The remaining authors declare that the research was conducted in the absence of any commercial or financial relationships that could be construed as a potential conflict of interest.

## Publisher's Note

All claims expressed in this article are solely those of the authors and do not necessarily represent those of their affiliated organizations, or those of the publisher, the editors and the reviewers. Any product that may be evaluated in this article, or claim that may be made by its manufacturer, is not guaranteed or endorsed by the publisher.
