# Supplementary material for: Pallidal Structural Changes Related to Levodopa-induced Dyskinesia in Parkinson's Disease
Source: Front Aging Neurosci. 2022 May 6;14:781883. doi: 10.3389/fnagi.2022.781883 (PMC9120819; doi:10.3389/fnagi.2022.781883)
Supplement: Supplementary file 1 [file Data_Sheet_1.docx]

Supplementary figure. The correlation result map between basal ganglia atrophy and the UDysRS score in each vertex. There was no vertex with significant relationship with dyskinesia severity.


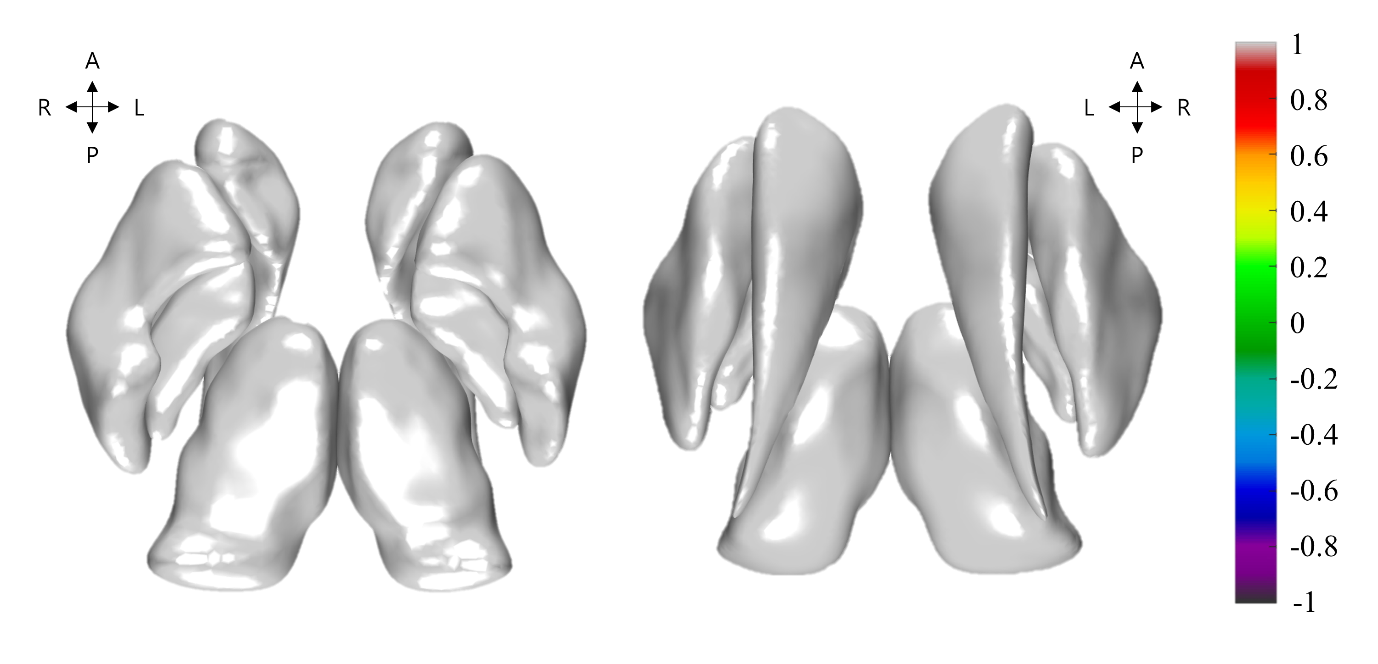


Supplementary table. Connectivity comparison results. The comparison was performed between four globus pallidus sub-regions and 85 brain regions. The P-values were calculated with two-sample t-tests and corrected based on Bonferroni correction.

| Globus pallidus  Right hemisphere | Left GPi | Right GPi | Left GPe | Right GPe | Globus pallidus  Left hemisphere | Left GPi | Right GPi | Left GPe | Right GPe |
| --- | --- | --- | --- | --- | --- | --- | --- | --- | --- |
| Lateral orbitofrontal | 1.000 | 1.000 | 1.000 | 1.000 | Lateral orbitofrontal | 1.000 | 1.000 | 1.000 | 1.000 |
| Pars orbitalis | 1.000 | 1.000 | 1.000 | 1.000 | Pars orbitalis | 1.000 | 1.000 | 1.000 | 1.000 |
| Frontal pole | 1.000 | 1.000 | 1.000 | 1.000 | Frontal pole | 1.000 | 1.000 | 1.000 | 1.000 |
| Medial orbitofrontal | 1.000 | 1.000 | 1.000 | 1.000 | Medial orbitofrontal | 1.000 | 1.000 | 1.000 | 1.000 |
| Pars triangularis | 1.000 | 1.000 | 1.000 | 1.000 | Pars triangularis | 1.000 | 1.000 | 1.000 | 1.000 |
| Pars opercularis | 1.000 | 1.000 | 1.000 | 1.000 | Pars opercularis | 1.000 | 1.000 | 1.000 | 1.000 |
| Rostral middle frontal | 1.000 | 1.000 | 1.000 | 1.000 | Rostral middle frontal | 1.000 | 0.134 | 1.000 | 1.000 |
| Superior frontal | 1.000 | 0.235 | 1.000 | 1.000 | Superior frontal | 1.000 | 1.000 | 1.000 | 1.000 |
| Caudal middle frontal | 1.000 | 1.000 | 1.000 | 1.000 | Caudal middle frontal | 1.000 | 1.000 | 1.000 | 1.000 |
| Precentral | 1.000 | 1.000 | 1.000 | 1.000 | Precentral | 1.000 | 1.000 | 1.000 | 1.000 |
| Paracentral | 1.000 | 1.000 | 1.000 | 1.000 | Paracentral | 1.000 | 1.000 | 1.000 | 1.000 |
| Rostral anterior cingulate | 1.000 | 1.000 | 1.000 | 1.000 | Rostral anterior cingulate | 1.000 | 1.000 | 1.000 | 1.000 |
| Caudal anterior cingulate | 1.000 | 1.000 | 1.000 | 1.000 | Caudal anterior cingulate | 1.000 | 1.000 | 1.000 | 1.000 |
| Isthmus cingulate | 1.000 | 1.000 | 1.000 | 1.000 | Isthmus cingulate | 1.000 | 1.000 | 1.000 | 1.000 |
| Postcentral | 1.000 | 1.000 | 1.000 | 1.000 | Postcentral | 1.000 | 1.000 | 1.000 | 1.000 |
| Supramarginal | 1.000 | 1.000 | 1.000 | 1.000 | Supramarginal | 1.000 | 1.000 | 1.000 | 1.000 |
| Superior parietal | 1.000 | 1.000 | 1.000 | 1.000 | Superior parietal | 1.000 | 1.000 | 1.000 | 1.000 |
| Inferior parietal | 1.000 | 1.000 | 1.000 | 1.000 | Inferior parietal | 1.000 | 1.000 | 1.000 | 1.000 |
| Precuneus | 1.000 | 1.000 | 1.000 | 1.000 | Precuneus | 1.000 | 1.000 | 1.000 | 1.000 |
| Cuneus | 1.000 | 1.000 | 1.000 | 1.000 | Cuneus | 1.000 | 1.000 | 1.000 | 1.000 |
| Perical carine | 1.000 | 1.000 | 1.000 | 1.000 | Perical carine | 1.000 | 1.000 | 1.000 | 1.000 |
| Lateral occipital | 1.000 | 1.000 | 1.000 | 1.000 | Lateral occipital | 1.000 | 1.000 | 1.000 | 1.000 |
| Lingual | 1.000 | 1.000 | 1.000 | 1.000 | Lingual | 1.000 | 1.000 | 1.000 | 1.000 |
| Fusiform | 1.000 | 1.000 | 1.000 | 1.000 | Fusiform | 1.000 | 1.000 | 1.000 | 1.000 |
| Parahippocampal | 1.000 | 1.000 | 1.000 | 1.000 | Parahippocampal | 1.000 | 1.000 | 1.000 | 1.000 |
| Entorhinal | 1.000 | 1.000 | 1.000 | 1.000 | Entorhinal | 1.000 | 1.000 | 1.000 | 1.000 |
| Temporal pole | 1.000 | 1.000 | 1.000 | 1.000 | Temporal pole | 1.000 | 1.000 | 1.000 | 1.000 |
| Inferior temporal | 1.000 | 1.000 | 1.000 | 1.000 | Inferior temporal | 1.000 | 1.000 | 1.000 | 1.000 |
| Middle temporal | 1.000 | 1.000 | 1.000 | 1.000 | Middle temporal | 1.000 | 1.000 | 1.000 | 1.000 |
| Bankssts | 1.000 | 1.000 | 1.000 | 1.000 | Bankssts | 1.000 | 1.000 | 1.000 | 1.000 |
| Superior temporal | 1.000 | 1.000 | 1.000 | 1.000 | Superior temporal | 1.000 | 1.000 | 1.000 | 1.000 |
| Transverse temporal | 1.000 | 1.000 | 1.000 | 1.000 | Transverse temporal | 1.000 | 1.000 | 1.000 | 1.000 |
| Insula | 1.000 | 1.000 | 1.000 | 1.000 | Insula | 1.000 | 1.000 | 1.000 | 1.000 |
| Thalamus | 1.000 | 1.000 | 1.000 | 1.000 | Thalamus | 1.000 | 1.000 | 1.000 | 1.000 |
| Caudate | 1.000 | 1.000 | 1.000 | 1.000 | Caudate | 0.017 | 1.000 | 1.000 | 1.000 |
| Putamen | 1.000 | 1.000 | 1.000 | 1.000 | Putamen | 1.000 | 1.000 | 1.000 | 1.000 |
| Globus pallidus externa | 1.000 | 1.000 | 1.000 | 1.000 | Globus pallidus externa | 1.000 | 1.000 | 1.000 | 1.000 |
| Globus pallidus interna | 1.000 | 1.000 | 1.000 | 1.000 | Globus pallidus interna | 1.000 | 1.000 | 1.000 | 1.000 |
| Accumbens area | 1.000 | 1.000 | 1.000 | 1.000 | Accumbens area | 1.000 | 1.000 | 1.000 | 1.000 |
| Hippocampus | 1.000 | 1.000 | 1.000 | 1.000 | Hippocampus | 1.000 | 1.000 | 1.000 | 1.000 |
| Amygdala | 1.000 | 1.000 | 1.000 | 1.000 | Amygdala | 0.125 | 1.000 | 1.000 | 1.000 |
|  |  |  |  |  | Brainstem | 1.000 | 1.000 | 1.000 | 1.000 |
